# Between Struggle and Strength: A Rapid Review of Dual-Trauma Couples

**DOI:** 10.1177/15248380251335036

**Published:** 2025-05-10

**Authors:** Véronique Charbonneau-Lefebvre, Marie-Pier Vaillancourt-Morel, Noémie Bigras, Eugenia Opuda, Ateret Gewirtz-Meydan

**Affiliations:** 1 Université du Québec à Trois-Rivières, QC, Canada; 2 Université du Québec en Outaouais, QC, Canada; 3 University of New Hampshire, Durham, NH, USA; 4 University of Haifa, Israel

**Keywords:** dual-trauma couples, couples, trauma, review, relationship dynamics

## Abstract

Although several studies have shown that one person’s trauma is associated with romantic relationship difficulties for both partners in adulthood, most have overlooked the particularity of dual-trauma couples, in which both partners have experienced traumatic experiences. This rapid review investigated the dynamics and challenges within dual-trauma couples. A rapid review methodology was employed to consolidate and evaluate existing knowledge in this field. Fifteen empirical research studies meeting the inclusion criteria were identified through a comprehensive search across multiple databases, including CINAHL, Family Studies Abstracts, PsycInfo, PubMed, Google Scholar, Web of Science, and Dissertations and Theses Global. Studies included encompassed different methodological approaches. The review focused on studies explicitly addressing dual-trauma couples, excluding those examining secondary trauma or broader impacts of trauma on couples without a specific analysis of dual-trauma dynamics. Key findings revealed a comprehensive understanding of the complexities faced by these couples, including relationship dynamics, communication patterns, psychological impacts of trauma, and factors influencing relationship satisfaction. The review showed that despite facing significant challenges, dual-trauma couples demonstrate remarkable strengths and resilience, emphasizing the importance of open communication and mutual support in coping with past traumas. The review underscored the need for tailored interventions and trauma-informed care to address the unique needs of dual-trauma couples. Through a focused exploration of existing literature, this review provides valuable insights for clinicians, researchers, and professionals working in the field of trauma and relationships, aiming to enhance understanding and support for these vulnerable couples.

## Introduction

The repercussions of trauma—defined as an event, a series of event, or a set of circumstances that is experienced as physically or emotionally harmful or threatening and that has lasting adverse effects on the individual’s functioning (SAMHSA, 2012)—are extensive and enduring. Trauma exposure may happen in childhood via the experience of various forms of childhood maltreatment or at any stage of life via, for instance, enduring life-threatening accidents or assaults, witnessing someone being injured or killed, sexual assault, and experiencing all kinds of natural disasters. While these experiences may all meet the diagnostic criteria for PTSD, their distinct origins can shape how trauma manifests in relationships. In a large national survey in the United States, 60.7% of men and 51.2% of women reported experiencing at least one trauma in their lifetime (Kessler et al., 1999, 2005).

Trauma exposure is known to affect the way survivors enter and experience romantic relationships (Campbell & Renshaw, 2018; Whisman, 2006). Initially, the literature on trauma and posttraumatic stress disorder (PTSD) focused on understanding its impact from an individual perspective. Results showed that trauma exposure is related to survivors’ difficulties in several aspects of romantic relationships, such as intimacy issues, intimate partner violence, sexual difficulties, and relationship dissatisfaction (Bergeron et al., 2022; Godbout et al., 2019; Vitek & Yeater, 2021). Subsequently, there was a notable shift toward recognizing the consequences of one’s traumatic experiences on both partners of the couple and addressing the secondary trauma experienced by partners of survivors (Dekel et al., 2016; Figley, 1995; Vaillancourt-Morel et al., 2023).

While efforts have been made to explore the effects of trauma on individuals and their partners (Finzi-Dottan & Gewirtz-Meydan, 2023; Vaillancourt-Morel et al., 2023), this evolving body of research has frequently overlooked the nuanced dynamics within dual-trauma couples—partnerships where both individuals have directly experienced trauma. Indeed, most past studies focused on how each partner’s trauma may affect the survivor, their partner, or the relationship, but the unique challenges and interactions within couples where both partners have directly experienced trauma have not been fully elucidated in the existing literature. This gap highlights the need for a more comprehensive examination of the specific dynamics inherent to dual-trauma couples, allowing for a more nuanced understanding of their shared experiences and relational complexities. This rapid review aims to unravel what the existing literature has to offer concerning dual-trauma couples and seeks to explore the depth of knowledge that has been accumulated in this field, to shed light on the dynamics, impacts, and interventions associated with these unique partnerships in which past traumas intersect with current relationships.

### Dual-Trauma Couples

Dual-trauma couples are defined as romantic partnerships in which both individuals have undergone traumatic experiences of any type (Balcom, 1996; Huber, 1997; Nelson et al., 2002). Given the magnitude of incidence rates of PTSD worldwide (Koenen et al., 2017), the likelihood of partners being involved in a dual-trauma couple is high. For instance, among a sample of 2,161 couples over the age of 50, 23.1% were dual-trauma couples (Whisman, 2014). The defining characteristic of dual-trauma couples is the simultaneous experience of trauma reactivity by both partners, setting them apart from single-survivors or non-traumatized couples (Nelson et al., 2002).

Several theoretical frameworks can be introduced to elucidate the formation of dual-trauma couples. It is possible that individuals who have yet fully addressed their own trauma tend to exhibit a proclivity for seeking partners who share similar unresolved issues. This inclination can be rooted in the solace they derive from knowing they are not alone in their struggles and that their partners can readily relate to their past experiences. A critical element in this dynamic is the role of attachment injuries. It is possible that these individuals actively seek partners capable of empathizing with their pain, effectively searching for individuals who can offer the understanding and emotional support that may have been absent during their own traumatic experiences (Scharff & Scharff, 2018). This shared empathy creates a distinctive sense of connection and mutual understanding within the relationship. Another potential explanation is the concept of repetition (Horowitz & Becker, 1971). It seems as though individuals are drawn to partners who unconsciously embody aspects of their prior experiences. Consequently, these individuals may find themselves reenacting familiar patterns or dynamics from their earlier life, even when those patterns were injurious or distressing (Macintosh, 2017). Finally, shared trauma histories may simply increase the likelihood of pairing, particularly among individuals with similar backgrounds, such as both partners serving in the military, residents of high conflict regions, or communities affected by natural disasters and collective historical trauma. These common experiences can create an initial bond that evolves into a romantic partnership.

In dual-trauma couples, both partners can experience heightened emotional reactivity, which can significantly affect the couple’s ability to develop and maintain intimacy. This situation requires therapists to focus on managing the intricacies of trauma responses within the relationship (Huber, 1997; Nelson et al., 2002). While empirical evidence has consistently linked trauma to various relational challenges (Campbell & Renshaw, 2018; Lambert et al., 2012; Sijercic et al., 2022), there is a lack of empirical studies to provide an adequate understanding of the intricacies of how these effects manifest in couples where both partners have experienced trauma. Therefore, the central question of the current review is how this double dose of trauma history influences the couple’s relationship. It can be inferred that dual-trauma couples are not solely shaped by their individual histories; rather, they function as an interactive unit. Understanding the dynamics of dual-trauma couples is crucial, as they represent potentially vulnerable couples in need of special support and services. Investigating how couples navigate trauma is a critical factor in comprehending the origins of relationship difficulties and identifying paths to alleviate dysfunction, reduce suffering, and enhance relationship satisfaction. By pinpointing specific areas where issues arise, such as failures in communication, misalignment between respective trauma coping strategies, or maladaptive attempts to fulfill each other’s needs, targeted changes can lead to positive outcomes (Fitzgerald & Shuler, 2022). Understanding these dynamics is essential because of the high prevalence of dual-trauma couples and because of the significant impact of trauma on relationship stability and individual well-being, which can be accelerated in dual-trauma couples.

### The Current Review

To date, the burgeoning research on the intersection of trauma and romantic relationships has mainly focused on examining trauma experiences in individuals, while examining its impact on both members of a romantic relationship. However, a critical gap exists in the literature concerning the nuanced dynamics, patterns, and outcomes within dual-trauma couples. To address this void, a rapid review is warranted to consolidate and evaluate existing knowledge on dual-trauma couples. This review aims to explore the depth of understanding accumulated in this field to shed light on the dynamics, impacts, and interventions associated with these unique partnerships. Through a focused exploration of the available literature, the rapid review intends to contribute to a more thorough understanding of dual-trauma couples, ultimately providing valuable insights for clinicians, researchers, and professionals working in the field of trauma and relationships.

## Method

### Types of Studies

This study aimed to identify empirical research focusing on dual-trauma couples. We conducted a comprehensive search for quantitative, qualitative, and mixed-methodology studies, including randomized controlled trials, phenomenological studies, surveys, and cohort studies. These studies were considered irrespective of whether they were conducted in clinical or community settings. To maintain a specific focus on dual-trauma couples, our review included only studies where both partners directly experienced trauma (i.e., primary trauma) and excluded those addressing secondary trauma (i.e., the emotional impact of indirect exposure to trauma), *such as cases where a person is affected by their romantic partner’s traumatic experiences without having personally experienced the trauma*. Additionally, studies that discussed the broader impact of trauma on couples and dual-trauma couples without specifically examining them in the context of couples (dyadic setting) were excluded.

### Participants

The study focused on dual-trauma couples, all of whom were over the age of 18. Trauma was broadly defined to encompass military-related trauma of any form (e.g., assault, injuries, threat to life, witnessing violent death, military sexual trauma), natural disasters, as well as relational trauma such as physical, sexual and/or emotional abuse or neglect from childhood to adulthood including PTSD and Complex PTSD as per DSM-5 criteria (American Psychiatric Association, 2013). The authors considered studies that combined reports from both single and dual-trauma couples, provided that the analysis clearly differentiated between the two groups. This inclusive approach aimed to capture a comprehensive understanding of trauma experiences within couples. Articles that did not differentiate between single and dual-trauma couples were excluded to ensure a more nuanced and specific exploration of the unique challenges faced by dual-trauma couples.

### Search Strategy

**Figure 1.**
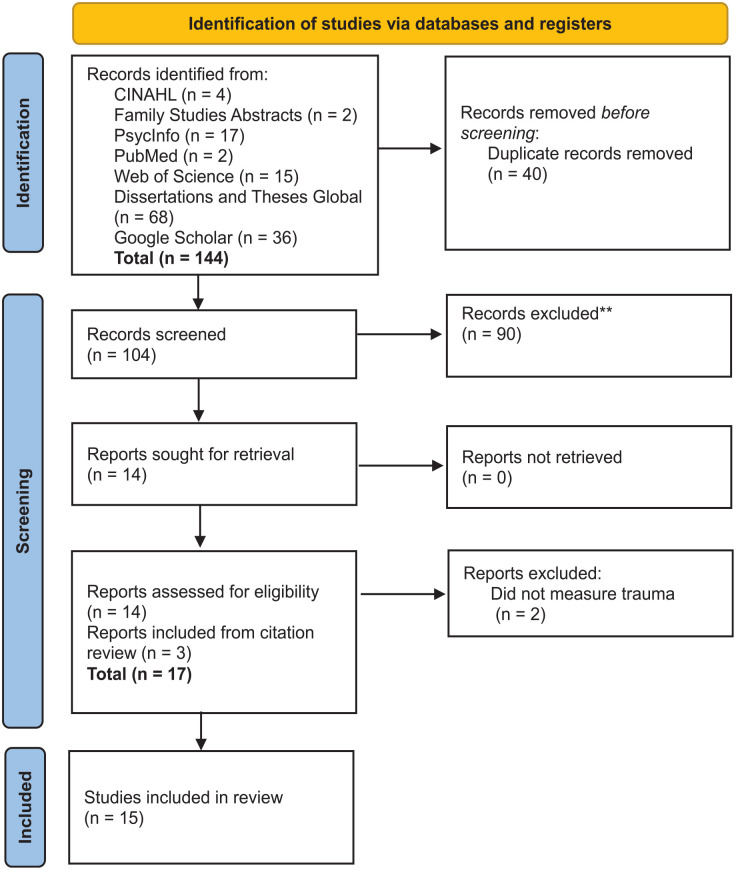
PRISMA 2020 Flow Diagram.

A keyword search for “Dual-trauma couples” served as the focal point in the search across seven databases, including CINAHL (EBSCO, 1937-current), Family Studies Abstracts (EBSCO), PsycInfo (EBSCO, 1800s-current), PubMed, Web of Science, and Dissertations and Theses Global (ProQuest, 1861-current). Relevant subject headings were not available that captured the definition of this term. Notably, gray literature databases were deliberately excluded, although instances of gray literature were identified within the database search. In tandem with the structured database search, a supplementary selection of citations from the Google Scholar search engine was incorporated for title and abstract review. The search did not include any language or publication date limitations, resulting in the identification of 144 records. After eliminating duplicates, 104 records underwent title and abstract screening, with 90 records failing to meet the inclusion criteria. This left 14 records for full-text screening. Three more records meeting the inclusion criteria were found through reviewing the citations of articles and were included in the review. The subsequent PRISMA flow diagram (Figure 1) visually delineates the inclusion and exclusion process, with 15 records deemed eligible for synthesis.

## Results

Overall, 15 studies that addressed dual-trauma couples were identified. Table 1 reports a summary of the studies included in the review, the methodology, sample size, setting of the study, and country. Table 2 presents the operative definitions of trauma and outcome measures, and Table 3 includes the main findings from each paper.

**Table 1.** Summary of Studies Included in the Review (*N* = 15).

Paper	Method	Sample size	Sample characteristics	Setting	Country
Alexander (2014)	Quantitative, Cross-sectional	*N* = 473 men who are court-mandated to treatment for intimate partner violence and their female partner	53 (11.2%) couples reported no trauma history, 93 (19.7%) couples reported men-only trauma history, 108 (22.8%) couples reported women-only trauma history, and 219 (46.3%) dual-trauma couples.	Self-reported intake questionnaire	USA
Banford Witting & Busby (2019)	Quantitative, Cross-sectional	*N* = 3,965 heterosexual couples	Physical violence: 1,991 couples reported no physical violence, 1,544 reported physical violence in one partner, and 472 couples both experienced physical violence. Sexual abuse: 2,678 couples reported no sexual abuse, 1,208 reported sexual abuse in one partner, and 121 couples both reported sexual abuse	Self-reported questionnaires	USA
Braughton et al. (2022)	Qualitative	*N* = 1,630 heterosexual dual-trauma couples	822 female and 831 male partners with a history of childhood exposure to physical, sexual and/or domestic violence	Self-report, open-ended questions	USA
Jackson (2023)	Qualitative	*N* = 5 heterosexual African America married couples	All (*N* = 5) dual-trauma couples	Semi-structured interview	USA
Lev-Wiesel & Amir (2003)	Quantitative, matched-pair design	*N* = 87 married couples	*N* = 43 Holocaust child survivors having experienced CSA, whose spouse was also a Holocaust child survivor who had experienced CSA compared with *N* = 44 survivors of CSA partnered had experienced non-CSA trauma during childhood	Self-report questionnaires	Israel
Nelson Goff et al. (2014)	Qualitative phenomenological study	*N* = 11 military couples	5 single-trauma couples and 6 dual-trauma couples	Semi-structured interview	USA
Nelson (1998)	Quantitative cross-sectional	*N* = 51 heterosexual couples seeking therapy	17 veteran couples, 17 CSA survivor couples, and 17 control no-trauma couples. Of the 51 couples, six (17.4%) were identified as dual-trauma couples, all composed of one veteran trauma individual and one CSA trauma survivor	Self-reported questionnaires through mail	USA
Nelson & Wampler (2000)	Quantitative, cross-sectional	*N* = 161 heterosexual couples seeking therapy	65 (40.3%) of couples reported no abuse, 24 (14.9%) couples reported male-only abuse, 57 (35.4%) couples reported female-only abuse, and 15 (9.3%) couples were dual-trauma couples.	Self-report questionnaires	USA
Nguyen et al. (2017)	Quantitative, observational, and longitudinal	*N* = 414 newlywed low-income heterosexual couples	24.6% of husbands and 31.1% of wives reported abuse as children. A total of 197 couples (45.7%) reported at least one type of abuse. 43 (10.4%) were dual-trauma couples.	Orally administered self-report questionnaires	USA
Redd (2017)	Quantitative cross-sectional	*N* = 146 sexually diverse couples presenting for couples therapy	50.7% female and 49.3% male participants	Self-report questionnaires	USA
Riggs (2014)	Quantitative cross-sectional	*N* = 50 male Vietnam combat veterans and their female romantic partner	52.0% of veterans and 28.0% of their female partners reported PTSD	Self-report questionnaires	USA
Ruhlmann et al. (2018)	Quantitative pilot study	*N* = 35 heterosexual married couples	18 dual-trauma couples (51.4%) and 17 single-trauma couples (48.6%)	Self-report questionnaires	USA
Shi (2020)	Quantitative cross-sectional	*N* = 107 heterosexual couples	All couples were dual-trauma couples	Self-report questionnaires	USA
Walker et al. (2011)	Quantitative cross-sectional	*N* = 10,061 heterosexual couples	7,255 couples (72.1%) were non-trauma couples, 2,539 (25.2%) were single-trauma couples and 267 (2.65%) were dual-trauma couples	Self-report questionnaires	USA
Whisman (2014)	Quantitative cross-sectional	*N* = 2,161 heterosexual couples over the age of 50	38.9% of women and 47.6% of men reported at least one trauma, and 23.1% were dual-trauma couples.	Self-report questionnaires	USA

**Table 2.** Operative definitions of trauma and outcome measures.

Paper	Type of trauma(s) examined	Main outcomes
Alexander (2014)	Physical and/or verbal violence perpetrated by either parental figure, experiences of sexual abuse perpetrated by any individual five years older or more, and exposure to intimate partner violence between parents before the age of 17 years old.	Reported by both partners: Intimate Partner Violence, including psychological aggression and physical assault. Reported by men only: Personality Assessment Inventory to measure affect regulation, violent behavior, and suicidal ideation; Alcohol Use Disorders Identification Test, to measure alcohol dependency, misuse, and social and legal issues related to excessive alcohol use; The Generality of Violence-Revised to measure a list of violent behaviors toward a list of eight category of people. Reported by women only: history of violence toward other women, history of arrests, tendency to get in physical fights, experiences of intimate partner violence in previous relationships, and total count of help-seeking strategies used for intimate partner violence, among the following : called the police, went to court, stayed at a shelter, called a hotline, talked to a lawyer, talked to clergy, saw a counselor, or attended group.
Banford Witting & Busby (2019)	Physical violence within the family and victimization, witnessing or perpetration of sexual abuse within the family or from someone outside the family, before the age of 18 years old.	The Negative Family Impact Scale to measure the impact of trauma on participants’ perception of relationships or their ability to form close relationships, the Negative Communication Scale to assess criticism, verbal disrespect and attacks, and highlighting faults during conflicts, the Calmness Scale to assess calmness and low anxiety as a form of a resource in participants, and the Relationship Instability Scale to measure the stability of participant’s relationship through time.
Braughton et al. (2022)	Childhood exposure to physical, sexual and/or domestic violence perpetrated by family members.	Open-ended questions examining couples’ strengths and weaknesses.
Jackson (2023)	Self-identified as having experienced at least one trauma experience.	7 open-ended semi-structured interview questions on their marital union, self and partner’s experience of trauma symptomatology, self and partner’s emotional needs, and marital satisfaction.
Lev-Wiesel & Amir (2003)	Self-reports of experiences of childhood trauma during interview by a trained social worker	Post-traumatic symptomatology (PTSD Questionnaire), general psychiatric symptomatology (Symptom Checklist 90 - Revised), and marital problems and strengths (Enrich Scale for Marital Quality)
Nelson Goff et al. (2014)	Nonmilitary trauma: childhood physical or sexual abuse, traumatic accident, natural disaster, victim of violent crime, violent sexual experiences in adulthood, physical abuse in adulthood, witnessing violent death, threat to life, news of death, other, or Military trauma: serving in war zone, threat to life, news of violent death, witnessing violent death. Measured with the Traumatic Events Questionnaire.	30 open-ended semi-structured interview questions focusing on previous deployment or trauma experiences, effects of their experiences on themselves or on their relationships, and dyadic adjustment.
Nelson (1998)	War trauma history or sexual abuse history on the Traumatic Events Questionnaires.	Trauma severity (Traumatic Events Questionnaires), post-traumatic stress symptoms (Purdue Post-Traumatic Stress Disorder Scale-Revised), secondary trauma symptoms (Secondary Purdue Post-Traumatic Stress Disorder Scale-Revised), general stress symptomatology (Brief Symptom Inventory), relationship quality (Dyadic Adjustment Scale), relationship satisfaction (Relationship Assessment Scale), and problematic dyadic interactions (Hostile/Avoidant/Supportive Couple Interactions Scale).
Nelson & Wampler (2000)	Experience of incest, physical childhood abuse, sexual childhood abuse, or unwanted sexual touching occurring before the age of 18 years old	Psychological distress (Brief Symptom Inventory), relationship quality (Dyadic Adjustment Scale), and family adjustment (Family Adaptability and Cohesion Scale)
Nguyen et al. (2017)	Experience of physical or sexual abuse before the age of 18 years old (categorical)	Relationship satisfaction (adapted measure) 2 years and 3 months after baseline
Redd (2017)	Childhood physical, psychological, and sexual abuse or neglect and exposure to household dysfunction (substance abuse, mental illness, suicide attempts, violence, family member going to prison, parental loss through abandonment, separation or divorce) prior to the age of 18, as measured by the Adverse Childhood Experiences questionnaire	Relationship quality (Dyadic Adjustment Scale – Revised) including consensus, satisfaction, and cohesion subscales.
Riggs (2014)	Post-traumatic stress disorder symptoms (Post-Traumatic Stress Disorder Checklist)	Relationship satisfaction (Dyadic Adjustment Scale), anxiety about close relationship with another (Fear of Intimacy Scale).
Ruhlmann et al. (2018)	Cumulative lifetime exposure to a variety of traumatic events measured by the Stressful Life Events Questionnaire	Marital satisfaction (Revised Dyadic Adjustment Scale) and perceived attachment behaviors (Brief Accessibility, Responsiveness, and Engagement Scale).
Shi (2020)	The Childhood Trauma Questionnaire was used to assess experiences of emotional and physical abuse or neglect, and sexual abuse before the age of 18 years old.	Trauma symptomatology including dysphoric mood, posttraumatic stress, sexual difficulties, and sense of self-dysfunctions (Trauma Symptom Inventory) and relationship satisfaction (Revised Dyadic Adjustment Scale).
Walker et al. (2011)	Childhood sexual abuse measured by experiences of sexual physical contact, abuse without physical contact or attempted sexual acts before the age of 18 years old	Self-report and partner contempt and defensiveness within romantic relationship
Whisman (2014)	Participants checked whether they had been in a major fire, flood, earthquake, or other natural disaster; had a life-threatening illness or accident; been a victim of a serious physical attack or assault; or been physically abused by either of their parents before the age of 18 years old	Marital quality was measured by questioning positive and negative marital interactions.

**Table 3.** Critical findings on dual-trauma couples.

Paper	Main results
Alexander (2014)	There were no differences in trauma severity between dual-trauma and single-trauma couples. Men in dual-trauma couples were significantly more likely to self-report physical and psychological aggression towards their partner than men in single-trauma couples or couples without a history of trauma. Men in dual-trauma couples reported greater antisocial personality traits than men in no-trauma couples and more suicidal thoughts, drug and alcohol abuse, and general violence than men without trauma, either in single- or no-trauma relationships. Partners of women with a history of trauma, either in single-trauma or dual-trauma couples, were more likely to report physical aggression from their female counterparts than men in no-trauma couples. Women in dual-trauma couples perceived themselves as being less likely to be physically aggressive than women in single-trauma or no-trauma couples. Women in dual-trauma couples were significantly less likely to describe their male partner as psychologically aggressive and equally likely than other women to describe them as being physically aggressive. They were also less likely to report their partner being arrested than women without trauma. They also described themselves as being less likely to engage in help-seeking behaviors.
Banford Witting & Busby (2019)	Women in dual-trauma couples reported greater negative relationship communication and lower relationship stability. Men in dual-trauma couples reported lower relationship stability.
Braughton et al. (2022)	Themes pulled from the qualitative data revealed that dual-trauma couples reported dyadic strengths that foster resiliency in their relationship, such as shared goals and beliefs, mutual collaboration, psychological flexibility, and feelings of connectedness. They also reported dyadic challenges regarding poor communication, disconnectedness, and difficulties with physical intimacy, emotional expression, distress tolerance, and relational safety.
Jackson (2023)	Dual-trauma African-American couples discussed themes of accommodation, attachment issues, loneliness, knowing each other, and loving and committing to their partner while describing their marital relationship and satisfaction. While discussing their trauma symptoms and their coping strategies, the themes emerging were safety, attachment, emotional needs, coping strategies, loneliness, and knowledge of triggers.
Lev-Wiesel & Amir (2003)	Individuals who were partnered with someone who had similar experiences of trauma (i.e., both CSA Holocaust survivors) reported greater PTSD symptoms, greater anxiety, somatization, hostility, depression and phobic anxiety than those who were with a partner with dissimilar trauma experiences, but they also reported greater marital quality than this last group.
Nelson Goff et al. (2014)	Both dual-trauma and single-trauma couples discussed higher awareness about the impact of trauma on their relationship, the demonstration of support from their partner, especially deployed soldiers, and positive and negative coping strategies while dealing with trauma were identified by couples. Dual-trauma couples discussed communication problems related to trauma and greater trauma triggers. For single-trauma couples, a unique theme that was reported consisted of having greater positive communication when discussing trauma-related issues.
Nelson (1998)	Veterans reported greater stress and trauma symptoms and greater trauma severity than CSA survivors or controls, and CSA survivors reported more trauma symptoms and severity, but not stress symptoms, than controls. Veteran partners reported greater stress symptoms and secondary trauma symptoms than CSA survivor partners or control partners. There were no significant differences in stress, trauma symptoms, secondary trauma symptoms, or trauma severity between CSA survivor partners and control partners. There were no significant differences in groups, nor for trauma-individuals or for their partner, in relationship functioning, namely relationship quality, satisfaction, and dyadic interactions. Contrary to the hypothesis, veterans in dual-trauma couples (i.e., partnered with a CSA-survivor) reported lower stress and trauma symptoms than veterans in single-trauma couples. CSA-partners in dual-trauma couples (i.e., partnered with a veteran) reported lower relationship quality than non-trauma partners of veterans.
Nelson & Wampler (2000)	Male partners reported significantly lower relationship quality in female-only trauma and dual-trauma couples compared to no-trauma couples. Female partners reported significantly lower relationship quality when in dual-trauma couples compared to no-trauma couples, but were no different than male-only or female-only trauma couples. Non-abused individuals in a relationship with an abused partner reported significantly greater levels of psychological distress. There were no significant differences in distress levels between dual-trauma couples and other groups. No differences were found between groups regarding family adjustment measures.
Nguyen et al. (2017)	Individuals with abuse history were more likely to marry someone who was also abused. Compared to husbands in dual-trauma couples, husbands without a history of abuse in a relationship with an abused wife reported lower relationship satisfaction. Abuse history was unrelated to the risk of divorce three years later. Husband’s abuse history was related to their own lower relationship satisfaction at baseline but was not significantly related to change in their relationship satisfaction through time. Wive’s abuse history was related to lower relationship satisfaction at baseline and a decline in their relationship satisfaction over 2.25 years.
Redd (2017)	Individuals’ greater experiences of adverse childhood experiences were associated with their own, but not their partners’, lower relationship quality. Couples were significantly more likely to match on their level of adverse childhood experiences than to differ, although this effect was small. There was a greater likelihood of similarity in couples with low scores of adverse childhood experiences than in couples with higher scores. The greater one’s experiences of adverse childhood experiences, the greater the odds of being partnered with an individual with minimal experiences of adverse childhood experiences, although this effect is small.
Riggs (2014)	Greater PTSD symptom severity in both veterans and their female counterparts predicted the couple’s lower relationship satisfaction score. Actors’ greater fear of intimacy acted as a mediator between one’s own greater PTSD symptom severity and the couple’s lower relationship satisfaction.
Ruhlmann et al. (2018)	In single-trauma couples, greater cumulative trauma experience in wives was associated with their husband’s lower marital satisfaction. In dual-trauma couples, greater cumulative trauma experience in wives was associated with their husbands’ greater perception of attachment-promoting behaviors (accessibility, responsiveness, and engagement) in their relationship. Greater PTSD symptoms in both wives and husbands were also associated with their own lower perception of attachment-promoting behaviors in their relationship. Greater PTSD symptoms in husbands were also associated with their own and their wives’ lower relationship satisfaction. The association between wives’ cumulative trauma exposure and husbands’ perceived attachment-promoting behavior and relationship satisfaction was moderated by the couple’s single or dual-trauma organization. Greater cumulative trauma exposure in wives was associated with a lessened perception of attachment-promoting behaviors and lower relationship satisfaction in husbands in single-trauma couples, and to a greater perception of attachment-promoting behaviors and greater relationship satisfaction in husbands in dual-trauma couples. The association between husbands’ PTSD symptomatology and their own relationship satisfaction was moderated by the couple’s single or dual-trauma organization, where husbands in single-trauma couples reported greater relationship satisfaction and husbands in dual-trauma couples reported lower relationship satisfaction.
Shi (2020)	In both men and women, one’s own trauma experiences, but not their partners’, were related to higher dysphoric mood and posttraumatic stress symptoms. In an opposite manner, in both men and women, sexual difficulties symptoms were related only to the partner’s trauma history. Sense of self-dysfunction was predicted by both self and partner’s experiences of trauma, for both men and women. Only women’s trauma score acted as a significant predictor of men’s relationship satisfaction.
Walker et al. (2011)	Non-abused individuals partnered with CSA-survivors reported being more contemptuous and defensive than individuals in a relationship with a non-abused partner. This effect was exacerbated in dual-trauma couples. Compared to no-trauma couples, couples with male-only reported abuse, male partners significantly perceived greater contempt and defensiveness in their partners. In dual-trauma couples, male partners also self-reported and perceived greater contempt and defensiveness than single-trauma and no-trauma couples.
Whisman (2014)	Individuals with a history of serious physical attack or assault, physical abuse as a child, or life-threatening illness reported lower marital quality. Lower marital quality was also reported by partners of individuals with a history of physical abuse as a child or serious physical attack or assault. Moderation analysis also shows that when both partner’s report experiences of serious physical attack or assault (i.e., dual-trauma couples), they report greater marital quality. A report of each trauma in one partner was significantly associated with an increased probability of the other partner reporting the same trauma, for all types of traumas.

### Methodological Characteristics

Studies were published between 1998 and 2023. Most studies were quantitative (*k* = 12), whereas three studies used a qualitative design. All studies used a retrospective design with adults, and almost all studies were cross-sectional (*k* = 14) except one longitudinal study with a 9-month, 18-month, and 27-month follow-ups. Sample size ranged from 5 to 10,061 couples (mean *N* = 1,290 couples; median *N* = 146), with a total of 38,714 participants (19,357 couples). All studies used a convenience sample of couples. Most couples were recruited in the community (*k* = 11), whereas 4 studies recruited clinical samples (i.e., couples seeking couples therapy, men court-mandated to treatment). Most studies included only mixed-sex couples (*k* = 12) (mostly described as heterosexual couples), two studies did not specify couple’s sexual orientation, and one study included mixed and same-sex couples. Most studies were conducted in the United States of America (*k* = 14), and one was conducted in Israel.

The findings from the 15 studies provide a comprehensive understanding of the dynamics and challenges faced by dual-trauma couples, highlighting both unique strengths and pervasive difficulties. The studies reviewed examined various types of traumas, including childhood physical, sexual, and emotional abuse, exposure to domestic violence, natural disasters, combat experiences, and life-threatening events (Alexander, 2014; Banford Witting & Busby, 2019; Nelson Goff et al., 2014; Shi, 2020; Whisman, 2014). The main outcomes assessed included intimate partner violence, relationship quality, satisfaction, communication patterns, trauma symptoms, and attachment behaviors (Alexander, 2014; Banford Witting & Busby, 2019; Nelson Goff et al., 2014; Redd, 2017; Shi, 2020; Whisman, 2014). While some studies focused exclusively on dual-trauma couples (e.g., Braughton et al., 2022; Jackson, 2023), most studies compared dual-trauma couples with single-trauma couples and/or no-trauma couples (e.g., Alexander, 2014; Bandford Witting & Busby, 2018; Nelson Goff et al., 2014). Additionally, a few studies did not create subgroups but instead examined the interaction between each partner’s trauma history to determine if the combination of both partners’ traumas has an effect beyond the effect of their individual trauma histories (e.g., Nguyen et al., 2017, Redd, 2017).

### Relationship Communication and Violence

Relationship communication and violence emerged as an area of concern for dual-trauma couples, across several studies, especially negative relationship dynamics and communication. For example, Alexander (2014) found that men in dual-trauma couples reported higher levels of physical and psychological violence perpetration compared to their counterparts in single-trauma or no-trauma couples. This aggression represents a negative relationship dynamic that often disrupts communication, as indicated by Banford Witting and Busby (2019), who noted that women in dual-trauma couples experienced more negative communication and lower relationship stability. Similarly, Walker et al. (2011) found that dual-trauma couples reported greater contemptuous and defensive communication patterns during conflict than single-trauma couples, which put couples at greater risk for dissatisfaction and dissolution. Nelson Goff et al. (2014) further highlighted that dual-trauma couples faced greater challenges with trauma-related communication issues and triggers, while single-trauma couples reported more positive communication when discussing trauma-related issues. In a qualitative study among 11 veterans and their female partners, dual-trauma couples shared some of their specific challenges, such as experiencing trauma-related triggers and significant communication difficulties. The study highlighted how both partners’ trauma histories often exacerbated these issues, creating a complex dynamic where mutual understanding and effective communication were hindered. The dual-trauma couples discussed their heightened sensitivity to triggers and the resultant strain on their interactions, which often led to misunderstandings and emotional disconnects (Nelson Goff et al., 2014).

### Psychological and Emotional Impact

The psychological and emotional impact of trauma on relationships was also noteworthy. Men in dual-trauma couples exhibited greater antisocial personality traits, suicidal thoughts, and substance abuse issues than those who had no trauma history (Alexander, 2014). Riggs (2014) reported that greater PTSD symptom severity in both veterans and their partners predicted lower relationship satisfaction, mediated by a fear of intimacy. This aligns with Shi (2020), who found that individuals’ own trauma experiences were linked to higher dysphoric mood and posttraumatic stress symptoms, affecting overall relationship dynamics and satisfaction in a sample of dual-trauma couples. Interestingly, Nelson and Wampler found greater levels of psychological distress only in non-abused individuals in single-trauma couples, compared to no-trauma couples, dual-trauma couples, suggesting that trauma may also impact the mental health of partners of survivors.

### Strengths and Resilience

Despite these challenges, dual-trauma couples demonstrated remarkable strengths and resilience. Braughton et al. (2022) identified strengths such as shared goals, mutual collaboration, and psychological flexibility, which fostered resilience in their relationships. Similarly, Jackson (2023) noted that dual-trauma couples expressed strong commitment and mutual understanding, despite facing significant emotional and attachment issues. In another qualitative study, trauma survivors and their partners provided insights into the intricate balance of positive aspects within dual-trauma couples. Despite the challenges posed by their shared trauma histories, dual-trauma couples demonstrated resilience and strength in their ability to navigate their experiences together. They emphasized the importance of open communication, mutual understanding, and supportive dynamics in coping with past traumas. This finding underscores the capacity of dual-trauma couples to leverage their shared experiences as a foundation for growth and mutual support within their relationship (Nelson Goff et al., 2014). While studies have yet to directly examine resilience in dual-trauma couples using validated measures, future research should prioritize this area to gain a deeper understanding of their strengths and vulnerabilities as their relationship unfolds over time.

### Relationship Satisfaction

The impact on relationship satisfaction varied significantly among dual-trauma couples. Nguyen et al. (2017) found that individuals with a history of abuse were more likely to marry someone with a similar history, and this shared trauma history was linked to lower relationship satisfaction over time. This can be attributed to the fact that trauma histories could inadvertently trigger and amplify each other’s trauma-related responses, resulting in an environment of constant emotional distress (Ruhlmann et al., 2018). Interestingly, Ruhlmann et al. (2018) observed that in dual-trauma couples, wives’ greater trauma exposure was associated with husbands’ greater perception of attachment behaviors, contrasting with single-trauma couples where wives’ trauma predicted lower marital satisfaction in husbands. This nuanced interplay suggests that shared trauma experiences can both challenge and strengthen relationship bonds. Emphasizing this finding, Lev-Wiesel and Amir (2003) found that although reporting greater PTSD symptoms and psychological distress, dual-trauma couples who had similar trauma experiences reported greater marital quality than dual-trauma couples who had dissimilar trauma experiences. Similar findings were reported by Whisman (2014), where dual-trauma couples with similar trauma experiences sometimes reported higher marital quality, indicating that shared experiences could foster greater understanding and connection. Finally, as found by Nelson and Wampler (2000) in their sample of couples seeking therapy, dual-trauma couples may have no greater relationship difficulties than single-trauma couples.

## Discussion

The findings of this review highlight the complex dynamics and challenges faced by dual-trauma couples, where both partners have experienced trauma. The review’s findings point to their challenges and difficulties, such as communication barriers, psychological distress, and varying levels of relationship satisfaction, but also uncovers their strengths, including resilience, mutual understanding, and shared coping mechanisms.

One of the most significant challenges facing dual-trauma couples is effective relationship dynamics and communication. Studies consistently reported the presence of aggression, negative communication patterns, and difficulties in discussing trauma-related issues and triggers (Alexander, 2014; Banford Witting & Busby, 2019; Nelson Goff et al., 2014). These communication barriers can exacerbate misunderstandings and emotional disconnectedness, further straining the relationship. Supporting this idea, other studies have found that trauma survivors tend to report more hostile and volatile conflict resolution strategies, significantly lowering relationship satisfaction and stability (Knapp et al., 2017; Orth & Wieland, 2006), which may also be the case in dual-trauma couples. On the other hand, open communication, mutual understanding, and supportive dynamics were identified as key strengths that fostered resilience in dual-trauma couples (Braughton et al., 2022; Jackson, 2023; Nelson Goff et al., 2014).

The challenges dual-trauma couples can face to communicate can be further exacerbated if they have experienced different types of trauma histories. The nature of the trauma experienced can influence its manifestation and impact on relationships. Interpersonal traumas, such as abuse, neglect, or domestic violence, often lead to attachment injuries, trust issues, emotional dysregulation, and a reluctance to be vulnerable with a partner (Riggs, 2014; Shi, 2021). In contrast, non-interpersonal traumas, like natural disasters or combat experiences, may result in survivor guilt, avoidance behaviors, and difficulties in sharing traumatic experiences due to the fear of not being understood (Nelson Goff et al., 2014). Dual-trauma couples may grapple with a combination of these complexities, as both partners navigate the unique challenges posed by their respective trauma types. Different trauma types can evoke varying responses from an individual’s support system, including their romantic partner. For instance, visible traumas—such as physical injuries from combat, violence, or loss caused by natural disasters—are often met with greater empathy and support. In contrast, less visible traumas, like emotional abuse or childhood neglect, may be overlooked, questioned by others, or even doubted by the survivor. Beyond visibility, there may be an unconscious collective hierarchy of trauma types (Ford et al., 2015), where some traumas are perceived as more severe or even noble, with these perceptions varying across cultures. While some studies suggest that specific trauma types are linked to a higher risk of PTSD (Conrad et al., 2017), other factors—such as subjective experience, cumulative exposure, timing, frequency, and severity—should also be considered when evaluating trauma’s impact (Gerke et al., 2018; Seguí-Grivé et al., 2024). The visibility and societal perception of an individual’s trauma type may influence not only how they cope with their trauma history within their relationship but also how they receive and offer support to their partner. These dynamics may shape psychological and relational outcomes, particularly in dual-trauma couples. In fact, one study found that, compared to dual trauma couples with dissimilar trauma experiences, dual-trauma couples having a similar trauma history reported greater psychological difficulties but also greater marital quality (Lev-Wiesel & Amir, 2003). Although only based on one study, this result highlights the need for further research on the interactions of individuals’ trauma histories when examining its impact on couple functioning.

Another critical point highlighted in this review is the profound individual psychological and emotional consequences that trauma experiences can have on both partners in dual-trauma couples. Studies found that men in these relationships exhibited higher rates of antisocial personality traits, suicidal thoughts, and substance abuse issues (Alexander, 2014). Furthermore, greater PTSD symptom severity, fear of intimacy, and dysphoric mood were associated with lower relationship satisfaction (Riggs, 2014; Shi, 2021). These severe psychological effects can create a cyclical pattern of distress, further exacerbating the already significant challenges faced by dual-trauma couples (Ein-Dor et al., 2010).

The findings on relationship satisfaction among dual-trauma couples were mixed and complex. While some studies reported lower relationship satisfaction (Nguyen et al., 2017; Ruhlmann et al., 2018), attributing it to the amplification of trauma-related responses and constant emotional distress, other studies suggested that shared trauma experiences could foster greater understanding, connection, and attachment behaviors, leading to higher marital quality in certain cases (Lev-Wiesel & Amir, 2003; Ruhlmann et al., 2018; Whisman, 2014).

It is possible that both the psychological impact and relationship satisfaction outcomes stem from a two-sided dynamic. On one hand, individuals who have not fully processed their own trauma may struggle to be emotionally available and supportive of their partner. They may unconsciously seek partners with similar unresolved issues, hence engage in repetition compulsion, in an attempt to resolve their own conflicts or gain a sense of mastery over their past traumas (Jackson, 2023). However, on the other hand, these individuals may also be drawn to partners who can empathize with their pain, finding solace in the shared understanding and emotional validation that may have been lacking during their traumatic experiences (Nelson Goff et al., 2014). This inclination could be driven by a need for emotional support and a sense of not being alone in their struggles.

Beyond these psychological mechanisms, the functioning of dual-trauma couples may also be impacted by key moderating and mediating factors, such as conflict resolution skills, social support, attachment security, and other resilience mechanisms (Baumann et al., 2024; Vaillancourt-Morel et al., 2021; Zamir, 2022). Effective conflict resolution strategies can serve as a protective factor, helping couples navigate trauma-related distress without escalating conflict or emotional withdrawal (Zamir et al., 2020). Similarly, social support—both within and outside the relationship—may buffer against the negative psychological and relational effects of trauma, reinforcing emotional stability and dyadic coping (Zamir, 2022, Zamir et al., 2018).

Shared beliefs and goals, mutual collaboration, psychological flexibility, and dyadic connectedness are some of the adaptive processes that enable dual-trauma couples to navigate the complexities of trauma-related experiences together (Braughton et al., 2022). However, barriers to resilience, such as individual perceptions, behaviors, and past experiences, can exacerbate relational instability and emotional unsafety (Braughton et al., 2022). For instance, a lack of effective conflict resolution skills may lead to maladaptive patterns, such as avoidance, blame, or emotional disengagement, which can further reinforce distress. Conversely, couples who actively cultivate resilience factors, including emotional regulation and shared meaning-making, may develop a stronger relational foundation despite the presence of trauma histories.

This dynamic could contribute to both psychological distress and the potential for deeper connection and understanding within the relationship. Thus, examining these moderating and mediating factors is crucial for identifying pathways that either hinder or enhance relationship functioning in dual-trauma couples (Zamir, 2022). These findings highlight the importance of considering both adaptive and maladaptive interactions in the conceptualization of trauma-affected couples, offering insights and directions for clinical treatment and future research.

It is this intricate interplay of factors – the shared empathy, the need for emotional validation, and the repetition of familiar patterns – that may also explain why strengths and resilience emerged as a prominent theme in some studies. Despite the significant challenges, dual-trauma couples demonstrated remarkable resilience, mutual understanding, and the potential for shared trauma to foster deeper connections (Braughton et al., 2022; Jackson, 2023; Nelson Goff et al., 2014). This highlights the complexity of the relationship between trauma experiences and couple functioning, where both vulnerabilities and strengths coexist.

The significance of studying dual-trauma couples lies in the profound impact trauma can have on romantic relationships. Trauma often leads to heightened emotional responses, communication difficulties, and challenges in maintaining relational stability. The presence of trauma in both partners can compound these issues, creating a complex interplay of individual and relational dynamics that necessitates specialized therapeutic approaches. Understanding these dynamics not only helps therapists in providing better care but also sheds light on the resilience processes that can be fostered within these couples (Table 4).

**Table 4.** Critical findings of the review.

Dual trauma couples encounter unique relationship dynamics and difficulties that remain largely unexplored by empirical studies, especially pertaining to communication, psychological distress, and relationship satisfaction.
Dual-trauma couples appear to present not only greater interpersonal challenges but also greater relationship strengths and resilience, compared to single or no-trauma couples.
The lack of longitudinal data prevents us from getting a prospective understanding of how these couples navigate long-term relationships.
It remains unclear whether different types of traumas (interpersonal trauma vs. non-interpersonal trauma, such as war or accidents) affect couples’ interpersonal functioning in different ways.

### Clinical Implications

The findings of this review offer insights into potential implications for clinical practice when engaging with dual-trauma couples. However, certain questions remain unanswered, prompting further consideration in this regard.

#### Couples Therapy or Not?

Given the complex dynamics at play, a crucial consideration arises regarding the suitability of couples therapy and whether the emotional reactivity and lack of responsiveness within the relationship may pose challenges in such a setting. The decision to engage in couples therapy should be carefully evaluated, considering the reciprocal effects of PTSD symptoms and the capacity of each partner to be emotionally present and responsive to the other. On one hand, the shared understanding and empathy that dual-trauma couples can provide for each other may serve as a strength and facilitate healing (Braughton et al., 2022; Jackson, 2023; Nelson Goff et al., 2014). However, if the emotional reactivity and distress within the relationship are too severe, and the partners are unable to be emotionally available or responsive to each other, couples therapy may not be the most appropriate approach. In situations where PTSD symptoms, such as emotional dysregulation, avoidance, or hyperarousal, significantly impair the ability of partners to engage in productive communication and emotional intimacy, individual therapy may be more beneficial initially (MacIntosh, 2019). By addressing individual-trauma processing and developing coping strategies, each partner may be better equipped to engage in couples work at a later stage.

#### Working with Different Types of Traumas

Another critical consideration is the potential challenge of working with couples who have experienced different types of traumas, particularly interpersonal and non-interpersonal traumas. As highlighted in the findings, these distinct trauma types can elicit unique challenges and responses, such as attachment injuries, trust issues, survivor guilt, and avoidance behaviors (Riggs, 2014; Shi, 2021; Nelson Goff et al., 2014). When working with dual-trauma couples, clinicians must be prepared to address the complexities that arise from the interplay of different trauma types. It may be necessary to dive into the specific nature of each partner’s trauma and explore how these experiences shape their individual and relational dynamics. However, clinicians must also be mindful of the potential for vicarious trauma and ensure that both partners have the emotional resources to engage with these sensitive topics without becoming overwhelmed. One approach may be to initially focus on building a secure therapeutic alliance and establishing a safe and supportive environment. This can involve validating each partner’s experiences, normalizing their reactions, and emphasizing the importance of self-care and emotional regulation. Once a foundation of trust and safety is established, clinicians can gradually explore the specific trauma narratives and their impacts on the relationship.

#### Tailored Interventions and Trauma-Informed Care

Regardless of the approach taken, it is crucial that interventions are tailored to the unique needs and experiences of dual-trauma couples. The treatment of dual-trauma couples has the intense challenges posed by these dyads, and patterns that complicate therapeutic processes, such as interpersonal reactivity, transference, emotional withdrawal, and associated responses (Balcom, 1996). Treatment approaches that focus on systemic influences of trauma are vital for these couples. Interventions need to challenge disruptive interactions, attend to the original traumas, and promote healthy relationship patterns. By addressing these aspects, therapists can assist dual-trauma couples in building more stable and supportive relationships despite their traumatic histories (Balcom, 1996). Nelson et al. (2002) emphasize the importance of employing a “trauma lens” in therapy to avoid overlooking critical cues about the systemic effects of traumatic stress. This approach helps therapists understand the unique dynamics of dual-trauma couples, which often feature marked interpersonal reactivity and conflict arising from each partner’s trauma narratives.

Trauma-informed interventions aim to address the psychological impact of trauma, particularly PTSD, depression, and anxiety, through approaches like cognitive-behavioral (couples) therapy (CBT), emotionally focused therapy (EFT), and eye movement desensitization and reprocessing (EMDR) treatment (Han et al., 2021; Linder et al., 2021; Taft et al., 2016, 2024). To date, research on couples-based PTSD interventions suggests that trauma-informed approaches yield the most significant improvements in reducing PTSD symptoms and contribute to enhancing relationship functioning (Sijercic et al., 2022), but the evidence supporting these interventions remains inconsistent. Research on dual-trauma couples is especially lacking, highlighting the need for further studies on how trauma-informed care can be adapted to relational dynamics and diverse trauma types, including interpersonal and collective trauma.

Additionally, clinicians should be prepared to address the specific challenges that dual-trauma couples may face, such as communication difficulties, emotional disconnection, and intimacy issues. MacIntosh (2019), in their couples therapy manual for complex trauma, highlights the importance of establishing foundational skills before directly addressing relationship difficulties and trauma. These foundational skills include mentalization, dissociation management, and emotional regulation, alongside psychoeducation on how trauma can impact intimacy, trust, communication, sexuality, and self-perception within the relationship. Interventions such as dyadic emotional coping exercises and breathing techniques help couples recognize maladaptive patterns, understand their links to trauma, and develop effective emotional regulation strategies to improve communication.

Ultimately, the decision to engage in couples therapy or pursue individual treatment should be made on a case-by-case basis, taking into account the severity of PTSD symptoms, the emotional resources of each partner, the specific dynamics and needs of the couple, and readiness for change. By focusing on the specific relational strengths and weaknesses of these couples, clinicians can better support dual-trauma couples in navigating their complex relational dynamics. This focus not only enhances therapeutic outcomes but also promotes resilience and stability for couples facing the dual challenges of their traumatic histories. Finally, therapeutic approaches for dual-trauma couples should not only focus on techniques, but also process factors, such as therapeutic alliance. Facilitating positive alliance is of foremost importance when working with people reporting PTSD symptoms, as relationship difficulties related to trust, self-esteem, power and control often play out in the therapeutic context (Taft et al., 2016).

### Limitations and Future Research Directions

While the present review provides valuable insights into the dynamics of dual-trauma couples, it is important to acknowledge several limitations and highlight areas for future research. All studies included relied on retrospective self-reported trauma histories, and almost all were cross-sectional. Thus, they could not provide information on causal associations between partners’ trauma and the couple functioning. Longitudinal studies tracking the trajectories of dual-trauma couples over time would be invaluable in understanding the long-term impacts of trauma on relationship dynamics, the efficacy of interventions, and the factors that contribute to resilience and positive adaptation.

Moreover, RCTs being crucial for determining best practices and evidence-based approaches tailored to the specific needs of these couples, future research should also prioritize conducting well-designed RCTs to compare the efficacy of various therapeutic modalities, such as couples therapy, individual-trauma-focused treatments, or integrative approaches. The generalizability of our results is potentially limited as all studies included used convenience samples of mixed-sex couples with low ethnic diversity, which significantly limits the generalization of the findings. While qualitative studies can provide rich insights into the lived experiences and narratives of dual-trauma couples, quantitative studies using larger and more representative samples can enhance statistical power and generalizability.

Another key limitation is the lack of in-depth information on the specific types of traumas experienced by couples in the reviewed studies. Additionally, the unique challenges and relational impacts associated with different trauma types were not thoroughly explored. Interpersonal traumas (e.g., childhood maltreatment, abuse, domestic violence), military-related trauma (e.g., combat exposure, life-threatening situations), and collective traumatic events (e.g., natural disasters) may affect romantic relationships in distinct ways. Future research should further investigate these nuances to better understand how various trauma experiences shape couple functioning and relationship dynamics. Moreover, most studies categorized couples into dual-trauma, single-trauma, or no-trauma couples based on a limited number of specific traumas, overlooking the severity and accumulation of diverse types of traumas. This categorization can be misleading as the “no-trauma couples” may not truly be without trauma or traumatic symptoms, and all traumatic histories may not have the same impact, considering that the severity, including the accumulation of diverse types of traumas is related to more negative outcomes. Advanced statistical methods (e.g., actor-partner interdependence model with the two-way interaction between each partner’s trauma, response surface analysis) could better account for the severity and range of trauma histories. Finally, when looking at the bigger picture, the most concerning limitation concerns the very minimal number of studies examining dual-trauma couples, where most research studying trauma, even when dyadic, overlooks the incidence of both partners having faced trauma and disregards the unique attributes and challenges dual-trauma couples face.

### Discussion of Diversity

Many of the reviewed studies were conducted in Western, Educated, Industrialized, Rich, and Democratic (WEIRD) countries, primarily in the United States. This limits the generalizability of the findings to more diverse cultural contexts and regions affected by conflict or war. As the prevalence of dual-trauma couples may vary across different sociocultural and geopolitical contexts, it is crucial to conduct research in non-WEIRD countries, particularly in regions where exposure to war-related trauma or societal violence is more prevalent. For instance, in the Middle East and other conflict-affected areas, the incidence of dual-trauma couples may be higher due to the increased likelihood of both partners experiencing war-related trauma, displacement, or exposure to violence. Moreover, couples living in high-conflict regions may rely more heavily on emotional suppression as a coping and survival mechanism, which can influence their relationship dynamics, communication patterns, intimacy, and problem-solving abilities in ways that differ from couples in WEIRD countries, highlighting the importance of conducting culturally sensitive research. Furthermore, trauma-related coping is often deeply influenced by sociocultural factors, including the stigma associated with mental health, the prevalence of community-based versus individualistic societal values, levels of religiosity, and other cultural norms, shaping the way trauma is reacted to, defined, perceived and treated (Ennis et al. 2020; Raghavan & Sandanapitchai, 2024). Future research should prioritize expanding the geographical and cultural diversity of study populations, including couples from diverse ethnic, religious, and socioeconomic backgrounds. By diversifying research samples and incorporating intersectional perspectives, researchers can gain a more comprehensive understanding of the challenges faced by dual-trauma couples across various cultural and societal contexts, ultimately improving the effectiveness of interventions and support services for these couples (Ennis et al. 2020).

Furthermore, most of the reviewed studies primarily focused on heterosexual couples, overlooking the experiences of same-sex dual-trauma couples. This omission is significant, as same-sex couples may face unique challenges related to their sexual orientation and gender identity in addition to their shared trauma experiences. Same-sex individuals often contend with societal stigma, discrimination, and marginalization, which can exacerbate feelings of isolation and psychological distress (Meyer, 2003). Given the intersectionality of trauma and minority stress, same-sex dual-trauma couples may face compounded difficulties in navigating their relationships and accessing appropriate support services. Research focusing on same-sex dual-trauma couples is essential for understanding how their experiences of trauma intersect with their identities as sexual and gender minorities, shaping their coping strategies, relationship dynamics, and overall well-being (Table 5).

**Table 5.** Implications of the review for practice, policy, and research.

A thorough assessment of couples’ psychological resources and coping mechanisms should be undertaken before recommending either individual or couples’ therapy
Interventions should be tailored to fit the unique complexities and needs of dual-trauma couples
Efforts in research should be deployed to determine factors explaining variations in relationship functioning (e.g., type of trauma, similar vs. dissimilar traumas between partners, couple configurations, cultural backgrounds, sex and gender diversity, etc.) in dual-trauma couples

## Conclusions

Findings from this rapid review collectively underscore the complex and multifaceted nature of dual-trauma relationships. While trauma significantly impacts communication, psychological health, and relationship satisfaction, many dual-trauma couples also exhibit resilience and strengths that help them navigate their challenges. Understanding these dynamics is crucial for developing targeted interventions and support mechanisms to help dual-trauma couples benefit from satisfying relationships and overall well-being.
